# Ultralow Charge Voltage Triggering Exceptional Post‐Charging Antibacterial Capability of Co_3_O_4_/MnOOH Nanoneedles for Skin Infection Treatment

**DOI:** 10.1002/advs.202207594

**Published:** 2023-01-26

**Authors:** Xianshuo Cao, Zongshao Li, Fan Yang, Jinhao Xie, Xin Shi, Peiyan Yuan, Xin Ding, Xihong Lu

**Affiliations:** ^1^ School of Chemistry School of Pharmaceutical Sciences (Shenzhen) The Key Lab of Low‐carbon Chem & Energy Conservation of Guangdong Province Sun Yat‐Sen University Guangzhou 510275 P. R. China

**Keywords:** antibacterial electrode, low charge voltage, post‐charging antibacterial, reactive oxygen species, wound infections

## Abstract

The post‐charging antibacterial therapy is highly promising for treatment of Gram‐negative bacterial wound infections. However, the therapeutic efficacy of the current electrode materials is yet unsatisfactory due to their low charge storage capacity and limited reactive oxygen species (ROS) yields. Herein, the design of MnOOH decorated Co_3_O_4_ nanoneedles (MCO) with exceptional post‐charging antibacterial effect against Gram‐negative bacteria at a low charge voltage and their implementation as a robust antibacterial electrode for skin wound treatment are reported. Taking advantaging of the increased active sites and enhanced OH^−^ adsorption capability, the charge storage capacity and ROS production of the MCO electrode are remarkably boosted. As a result, the MCO electrode after charging at an ultralow voltage of 1.4 V gives a 5.49 log and 5.82 log bacterial reduction in *Escherichia coli* (*E. coli*) and *Pseudomonas aeruginosa* (*P. aeruginosa*) within an incubation time of only 5 min, respectively. More importantly, the antibacterial efficiency of the MCO electrode against multi‐drug resistant (MDR) bacteria including *Klebsiella pneumoniae* (*K. pneumoniae*) and *Acinetobacter baumannii* (*A. baumannii*) also reaches 99.999%. In addition, the MCO electrode exhibits excellent reusability, and the role of extracellular ROS in enhancing post‐charging antibacterial activity is also unraveled.

## Graphical abstract

MnOOH decorated CO_3_O_4_ nanoneedles (MCO) capable of exceptional post‐charging antibacterial effect against Gram‐negative bacteria at a low charge voltage are designed. The decoration of MnOOH not only significantly boosts the charge storage capacity of nanoneedles by providing abundant electrochemical active sites, which will disturb the respiration process of bacteria and induce the intracellular ROS burst, but also dramatically increases the extracellular ROS production by reducing the adsorption energy of OH^−^. As a result, the combination of extracellular ROS and intracellular ROS shows enhanced antibacterial activity both in vitro (>99.999%) and in vivo (96%).

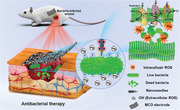

## 1 Introduction

Bacterial infections occurring in the wound healing process remain one of the most serious public safety issues because of its high morbidity and mortality.^[^ ^1^, ^2^, ^3^, ^4^ ^]^ Antibiotics have been recognized as the most common treatment for bacterial infections, but the efficiency of antibiotics is threatened by the increased antibiotic resistance.^[^ ^5^, ^6^, ^7^, ^8^, ^9^, ^10^ ^]^ In particular, Gram‐negative bacteria such as *E. coli*, *K. pneumoniae*, *A. baumannii*, and *P. aeruginosa* are listed as the “critical priority pathogens” by World Health Organization.^[^ ^11^, ^12^ ^]^ These bacteria are extremely difficult to treat, as they show high level of antibiotic resistance and low drug permeability due to the double‐layer membrane structure.^[^ ^13^, ^14^, ^15^ ^]^ For instance, the multidrug resistant *E. coli* bacteria with mcr‐1 genes is resistant to colistin, an antibiotic which is the last choice for treating multi‐drug resistant (MDR) bacterial infections.^[^ ^16^ ^]^ With the pipeline of new antibiotics drying up, it is urgent to develop a safe, effective and non‐antibiotic approach to conquer Gram‐negative bacterial infections and prevent resistance development.

Over the past few decades, photodynamic therapy (PDT),^[^ ^17^ ^]^ photo‐thermal therapy (PTT),^[^ ^18^ ^]^ sonodynamic therapy (SDT),^[^ ^19^ ^]^ macromolecular antimicrobials^[^ ^20^, ^21^, ^22^, ^23^ ^]^ with non‐antibiotic strategies have been reported in treating wound infection. Among current antibacterial methods, posting‐charging antibacterial approach has shown distinct promise to combat bacterial infections due to their high killing efficiency, rapid antibacterial ability, noninvasive and effectiveness in preventing drug resistance development. For instance, Tian et al. observed that the ZnO/Ag electrodes powered by triboelectric nano‐generator showed instant and sustained antibacterial effects for various microbes.^[^ ^24^ ^]^ A post‐charging antibacterial activity of 6 log reduction in *E. coli* was attained at a high output voltage of 50 V. Recently, Wang's group constructed a carbon doped TiO_2_ nanotube arrays on the Ti substrates to reduce the voltage required for antimicrobial activity, which achieved a post‐charging antibacterial activity against *E. coli* (80%) at 2 V.^[^ ^25^ ^]^ Although those encouraging processes, due to small charge storage capacity limited by their double‐layer mechanism, the post‐charging antibacterial activity of these electrode materials is yet unsatisfactory, particularly at low charging voltage. Another issue of these antibacterial materials for skin infections treatment is that their current collectors are not flexible, which is not desirable for skin wound with different shapes.^[^ ^26^ ^]^ Thus, exploring new electrode with excellent post‐charging antibacterial activity and flexibility is still challenging but highly benefit for clinical translation.

**Scheme 1:**
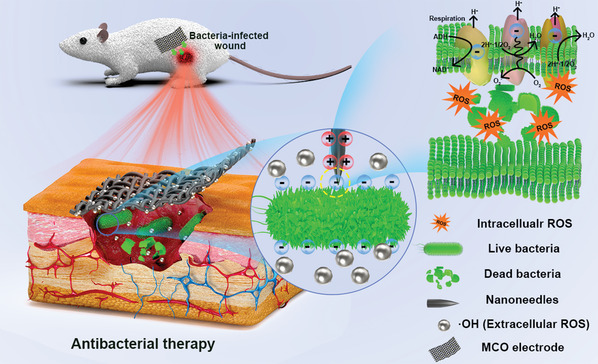
Schematic diagram for the antibacterial mechanism of MCO.

Cobaltosic oxide (Co_3_O_4_) is a highly desirable redox electrode material for energy storage due to its multiple valence states and exceptional electrochemical activity.^[^ ^27^, ^28^ ^]^ Recently, a Co_3_O_4_ nanowire electrode after charging at 2 V for 30 min exhibited a good antibacterial rate against *E. coli* (≈93.8%, 1 log reduction) within 30 min treatment, showing the great potential of Co_3_O_4_ as promising post‐charging candidate for bacterial infection therapy.^[^ ^29^ ^]^ However, the critical issue for Co_3_O_4_ is that its antibacterial efficacy is still low owing to its insufficient active sites and limited ROS yield. In this work, we remove these roadblocks by decorating Co_3_O_4_ (CO) nanoneedles grown on the flexible carbon cloth with a thin MnOOH nanoparticles, which remarkably boost their post‐charging antibacterial activity (**Scheme** **1**). The decoration of MnOOH not only significantly boosts the charge storage capacity of CO nanoneedles by providing abundant electrochemical active sites, but also dramatically increases the extracellular ROS production by reducing the adsorption energy of OH^−^. As a result, a robust post‐charging antibacterial activity with 5.49 and 5.82 log reduction in the cell number of *E. coli* and *P. aeruginosa* respectively within a very short time treatment (only 5 min) is achieved for MnOOH decorated Co_3_O_4_ nanoneedles (MCO) electrode after charging at an ultralow voltage of 1.4 V for 30 min. Moreover, this MCO electrode also delivers outstanding antibacterial activity of 5.85 and 5.19 log reduction in *K. pneumoniae* and *A. baumannii*, respectively. Further experimental results confirm that the MCO electrode possesses stable antibacterial activity and its enhanced post‐charging antibacterial performance is mainly due to the synergistic effect of the intracellular and extracellular ROS. Additionally, a mouse skin infection model in vivo therapeutic results reveal that the MCO has excellent in vivo antibacterial rate and is able to dramatically promote wound healing.

## 2 Results

**Figure 1:**
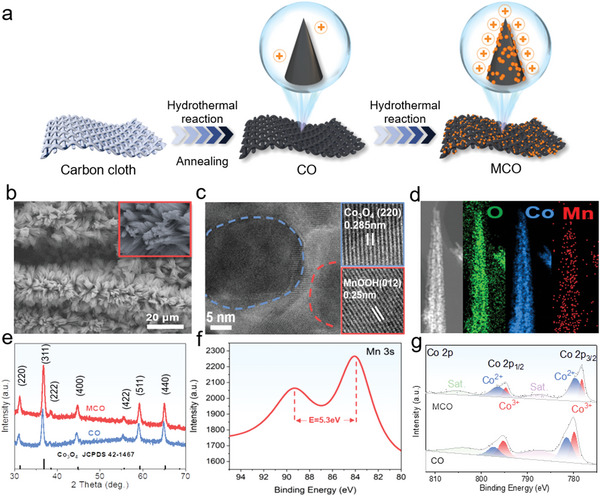
a) Illustration of the synthetic process of MCO, b) SEM, c) HRTEM, d) Element mapping, e) XRD patterns of CO and MCO. f) Mn 3s core‐level XPS spectra of MCO. g) Co 2p core‐level XPS spectra for CO and MCO.

The fabrication process of MCO electrode is shown in (**Figure** **1**a). Typically, the cobalt‐based precursor was directly grown on the carbon cloth fibers via a simple hydrothermal method, and the precursor was converted into CO nanoneedles by an annealing process in air. After that, MnOOH was coated onto the surface of CO nanoneedles through a hydrothermal treatment to form MCO. The as‐prepared MCO grows uniformly upon the carbon cloth fibers from the results of scanning electron microscopy (SEM) image of MCO (Figure 1b). Furthermore, the needle‐like profile of the electrode is clearly observed in macro image (inserted in Figure 1b), which may enhance the electric field effect for sterilization. The high resolution transmission electron microscopy (HRTEM) image confirms that the nanoneedles are highly crystalline. Additionally, an inter‐planar spacing of 0.285 and 0.25 nm corresponding to the (220) and (012) plane of Co_3_O_4_ and MnOOH are clearly identified, demonstrating the successful recombination of Co_3_O_4_ and MnOOH (Figure 1c). From the energy dispersive spectroscopy (EDS) mapping images (Figure 1d), the Co, Mn and O elements are evenly distributed in the nanoneedles, further proving the successful decoration of MnOOH onto the Co_3_O_4_ nanoneedles surface.

The crystal phases and chemical composition of the obtained CO and MCO were investigated by X‐ray diffraction (XRD) and X‐ray photoelectron spectroscopy (XPS). The characteristic peaks of both samples can match well with the cubic Co_3_O_4_ (JCPDS 42‐1467, Figure 1e). In detail, the characteristic peaks located at 31.2°, 36.8°, 44.8°, 55.6°, 59.3°, 65.2° are ascribed to the (220), (311), (400), (422), (511), and (440) planes of Co_3_O_4_. The MnOOH diffraction peaks are not directly detected in XRD patterns due to the low contents of MnOOH (3.52%) from the EDS spectrum analysis (Figure S1, Supporting Information). However, the typical characteristic peaks of MnOOH are observed in the XRD patterns of the precipitation generated during the MCO synthesis, confirming that the presence of MnOOH in MCO (Figure S2, Supporting Information). The full XPS survey spectra of CO and MCO clearly reveal the existence of Mn element in MCO (Figure S3, Supporting Information). For MCO, the Mn 2p peak displays the core‐level Mn 2p_3/2_ and Mn 2p_1/2_ peaks at binding energies of 642.3 and 653.9 eV (Figure S4, Supporting Information), which are the typical characteristic peaks of Mn^3+^.^[^ ^30^, ^31^ ^]^ Moreover, the binding energy difference of 5.3 eV in Mn 3s spectrum further confirms the main valence state of Mn is +3 (Figure 1f).^[^ ^32^ ^]^ The high‐resolution Co 2p spectrum can be deconvoluted into six peaks corresponding to the spin−orbit of Co 2p_3/2_ and Co 2p_1/2_ and their satellite peaks (Figure 1g). In the high‐resolution Co 2p spectrum of CO, two main peaks of centered at 780.18 and 795.48 eV are assigned to the Co 2p_3/2_ and Co 2p_1/2_ spin−orbit peaks, respectively. However, the Co 2p_3/2_ and Co 2p_1/2_ of MCO shift to a lower binding energy of 779.88 and 794.88 eV, indicating that the Co^2+^‐rich surfaces are formed due to the intensive interfacial electron interaction between Co_3_O_4_ and MnOOH.

**Figure 2:**
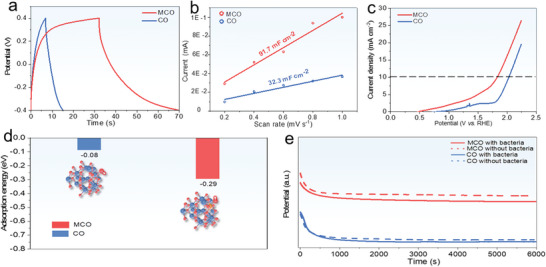
a) GCD curves of different electrodes. b) The ratio of current density with different scan rate. c) Liner sweep voltammetry curves of different electrodes. d) The OH^−^ adsorption energy of different electrodes and the corresponding atomic models. e) The potential curves of different electrodes.

The charge storage capability of as‐prepared samples was firstly evaluated by using cyclic voltammetry (CV) method. The MCO possesses a larger area enclosed by the CV curve, implying its boosted charge storage capacity after MnOOH decoration (Figure S5, Supporting Information). Galvanostatic charge/discharge (GCD) curves of both samples are further compared in **Figure** **2**a. The MCO displays a longer discharge time, again confirming that the MnOOH decoration can significantly enhance the charge storage capacity. In addition, the electrochemical impedance spectroscopy (EIS) was conducted to analyze the mass transport kinetics of the samples. The semicircle diameter in Nyquist plots represents the charge transfer resistance (*R*_ct_) (Figure S6, Supporting Information).^[^ ^33^, ^34^ ^]^ Obviously, there is no significant difference in *R*_ct_ between two samples, uncovering that the decoration of MnOOH has almost no effect on electrical conductivity. To understand the improved charge storage capability of MCO, the electrochemical surface areas (ECSA) of both electrodes were estimated through the identification of double‐layer capacitance (*C*_dl_) measured by collecting CV curves at different scan rates (Figure 2b). Unambiguously, the MCO delivers a larger *C*_dl_ value (91.75 mF cm^−2^), demonstrating that the MnOOH decoration can substantially enrich accessible active sites, thereby boosting the charge storage capacity. Additionally, the amount of oxygen molecules upon electrolyze water process is positively correlated with the amount of •OH, generally the more oxygen molecules produce, the more •OH will be generated.^[^ ^35^, ^36^, ^37^ ^]^ The oxygen evolution activity of CO and MCO electrodes is compared (Figure 2c). The over‐potential of the MCO electrode at 10 mA cm^2^ is about 598 mV, considerably smaller than the CO electrode (798 mV), confirming its superior oxygen evolution activity. This also indicates that the introduction of MnOOH can facilitate the production of •OH. For better uncovering the effect of MnOOH, density functional theory (DFT) calculation was performed. The adsorption step of OH^–^ is the limited step of the •OH production during oxygen evolution process.^[^ ^38^ ^]^ The calculated OH^–^ adsorption energy for MCO electrode (−0.29 eV) is far lower than the CO electrode (−0.08 eV), manifesting that the OH^–^ adsorption capability of CO is obviously enhanced after MnOOH decoration and will promote the formation of •OH (Figure 2d). We also analyze the interaction between bacteria and electrodes by open circuit potential (OCP) test since the potential will change as a result of charge transfer when the bacteria and electrode touch each other. A significant potential drop is observed for MCO electrode after adding the *E. coli* into the electrolyte (Figure 2e), verifying the MnOOH decoration can significantly enhance the interfacial interaction between MCO electrode and bacteria, thus endowing it has great antibacterial potential.

**Figure 3:**
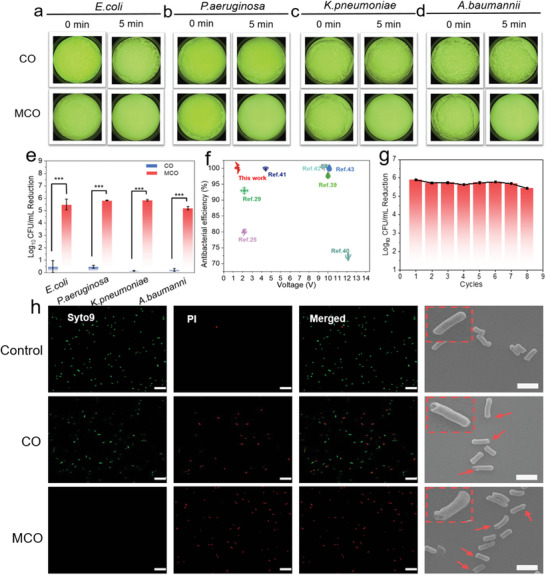
a–d) Representative plate colony images of the *E. coli*, *P. aeruginosa*, *K. pneumoniae*, and *A. baumannii* (10^6^ CFU mL^−1^) exposure of different electrodes at 1.4 V for 5 min. e) Antibacterial activity of *E. coli*, *P. aeruginosa*, *K. pneumoniae*, and *A. baumannii* treated with different electrodes (*n* = 3, ***p *<* 0.01). f) Summary of the antibacterial performance of MCO and other recently reported electrode against *E. coli*. g) The post‐charging antibacterial stability of the MCO against *E. coli*. h) The corresponding live/dead staining (scale bar = 10 µm) and SEM images of *E. coli* treated with different electrodes (scale bar = 2 µm).

The post‐charging antibacterial activity of MCO electrode under different voltages against *E. coli* was firstly evaluated via plate counting methods. When the voltage of 1.4 V is applied, the MCO started to show significant antibacterial activity (Figure S7, Supporting Information). Therefore, the charge voltage is fixed at 1.4 V for subsequent experiments. It should be pointed out that both the pristine MnOOH and uncharged MCO electrode presents no significant antibacterial activity (Figures S8 and S9, Supporting Information). Compared with CO groups, the culture plates for the *E. coli* show no colonies after treatment with MCO electrode for 5 min (**Figure** **3**a), confirming its outstanding antibacterial effect. More importantly, this MCO electrode also possesses superb antibacterial activity against other Gram‐negative bacteria such as *P. aeruginosa*, *K. pneumoniae*, and *A. baumannii* (Figure 3b–d), which the latter two are the typical MDR bacteria. The antibacterial efficiency of MCO electrode is determined to be >99.999% (>5 log bacterial reduction) for all the bacteria tested, while the antibacterial efficiency of CO electrode against *E. coli*, *P. aeruginosa*, *K. pneumoniae*, *A. baumannii* only attains to 0.48, 0.47, 0.14, and 0.21 log reduction, respectively (Figure 3e). Additionally, to our knowledge, the antibacterial performance against *E. coli* is the best reported to date for electrical antibacterial (Figure 3f).^[^ ^25^, ^29^, ^39^, ^40^, ^41^, ^42^, ^43^ ^]^ More importantly, the MCO still maintains exceeding antibacterial stability even after 8 cycles of charging–discharging (Figure 3g).

To further investigate the post‐charging antibacterial activity, the live/dead bacterial staining and SEM observation were carried out. The *E. coli* treated by MCO presents strong red fluorescence, suggesting that almost all the bacteria are dead after discharging treatment (Figure 3h). However, only a few red fluorescence is observed in CO treatment group. Noticeably, the same trend of fluorescence test and plate counting further verify that the decoration of MnOOH can improve post‐charging antibacterial activity. The morphology changes of *E. coli* after different treatments were also evaluated by SEM. Compared to untreated *E. coli*, some pores (marked by red arrow) are appeared in the *E. coli* treated by CO and MCO electrode (Figure 3h), indicating that the membrane can be destroyed by discharging treatment. Furthermore, this MCO electrode exhibits excellent biocompatibility (Figure S10, Supporting Information), which implies it can specifically kill bacteria without toxic effect on normal skin cells.

**Figure 4:**
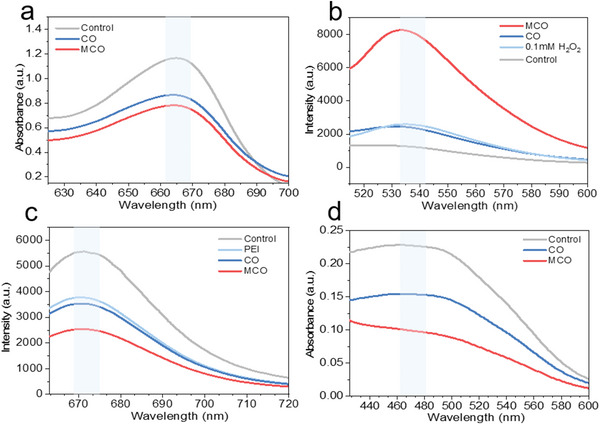
a) Extracellular ROS (•OH) generation characterized by the UV–vis absorption spectra of MB. b) The fluorescence spectra of *E. coli* stained with intracellular fluorescent ROS probe DCFH‐DA. c) The change of cell membrane potential reflected by the intensity of DiSC3 (5) incubated with *E. coli* treated with different electrodes. d) UV–vis absorbance of INT to monitor respiration of *E. coli* treated with different electrodes.

To unravel the mechanism of the improved post‐charging antibacterial performance, the expression of extracellular ROS, including •OH and ^1^O_2_, was investigated using Methylene blue (MB) and 1,3‐diphenylisobenzofuran (DPBF) as the trapping agent, respectively. The absorption intensity of MB is significantly decreased in the presence of MCO (**Figure** **4**a). However, there is no significant difference in the adsorption intensity of DPBF for all samples (Figure S11, Supporting Information). It is inferred that •OH is the primary extracellular ROS generated by MCO during charging. When exposed to bacterial solution, these •OH adsorbed on the surface of electrodes are released to perform the first antibacterial action. The intracellular ROS treated with different electrodes was measured using DCFH‐DA as an indicator. The bacteria treated by MCO exhibit higher fluorescent intensity compared to control and positive group (Figure 4b). Moreover, the confocal images of bacteria further verify the expression of intracellular ROS, and those treated with MCO present brighter fluorescence (Figure S12, Supporting Information). The ROS could come from respiration chain that had been disturbed by the electrons. The respiration is a universal behavior in bacteria that involved in electron transport. The electrons produced by oxidation of organics for adenosine triphosphate generation are transmitted to O_2_, in which ROS is produced by partial O_2_ reduction under normal circumstances. However, the external charge could disturb the respiration, resulting in ROS burst and bacteria death.^[^ ^24^ ^]^ To make clear whether the respiratory chain is disrupted when the bacteria contact with the electrodes, the changes in bacterial membrane potential and respiratory chain were investigated. The cell membrane potential as external manifestation of charge transfer occurred in respiratory chain was first studied. The florescence intensity dramatically is decreased after MCO treatments in comparison to CO (Figure 4c), suggesting that the respiratory chain is affected by disturbing charge transfer during post‐charging anti‐bacteria process. Furthermore, we used a reduction‐sensitive dye named iodonitrotetrazolium chloride (INT) to monitor bacterial respiration (Figure 4d). A significantly decreased bacterial respiration treated by MCO is observed, which confirms that the respiration chain is disrupted indeed. Overall, the improved antibacterial activity of MCO is due to the synergistic effect of extracellular ROS and intracellular ROS.

**Figure 5:**
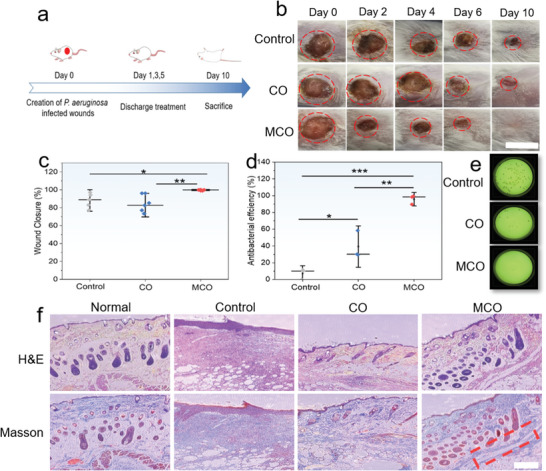
a) Schematic illustration of procedures on in vivo discharging against bacteria infected mouse skin wound model. b) Photographs of *P. aeruginosa* infected wounds treated with different samples from day 0 to day 10 (scale bar: 1 cm). c) Quantitative analysis of wound closure after 10 days (*p *<* 0.1, **p < 0.05, ***p *<* 0.01). d) Comparisons on the antibacterial efficiency after 6‐day treatments in different groups (*p *<* 0.1, **p < 0.05, ***p *<* 0.01). e) Representative bacteria colony of the skin tissue acquired from wound after 6 days of infection. f) H&E staining and Masson's trichrome staining of the skin tissues (scale bar: 100 µm).

The in vivo antibacterial therapeutic effect of MCO was explored in a *P. aeruginosa* infected Balb/C mice skin wound models. The randomly grouped animals are subjected to a treatment schedule depicted in **Figure** **5**a. Compared with control or CO group, the wound of infected mice treated with MCO are effectively healed without obvious scar in 10 days (Figure 5b). However, the larger wound area and significant scar still can be visualized at the control or CO group after 10 days, indicating the MCO exerts excellent wound healing effect. The quantitative assessment of wound closure shows that the mice treated with MCO display average percentage 99% wound closure after 10 days (Figure 5c), which is significantly higher than the control (88%) or CO (82%) group (*p* < 0.05). The fast wound healing is mainly attributed to the in vivo antibacterial effect of MCO. Compared to the control group (7%), only 39% killing rate is achieved for the CO‐treated mice. In contrast, the MCO displays excellent in vivo bacteria killing effectiveness, and almost all bacteria in wound are eradicated on Day 6 (96%, Figure 5d,e). The hematoxylin and eosin (H&E) and Masson staining was conducted to confirm the wound healing effect (Figure 5f). For MCO group, no evident inflammation occurs, and the new blood vessels are detected (marked by red box). In contrast, the infiltration of many inflammation cells appears in the control or CO group. These results confirm that MCO is a promising electrode for in vivo antibacterial treatment.

## 3 Conclusion

In summary, we successfully construct a MnOOH decorated Co_3_O_4_ nanoneedle electrode and achieve effective electric antibacterial therapy of Gram‐negative bacteria infected skin wound. By virtue of incremental active sites and optimized OH^−^ adsorption capability, the MCO electrode exhibits substantially improved charge storage capability and ROS yields, and thus giving a >5 log bacterial reduction in typical Gram‐negative bacteria (*P. aeruginosa* and *E. coli*) and MDR Gram‐negative bacteria (*K. pneumoniae* and *A. baumannii*) at a low voltage of 1.4 V. Moreover, this MCO electrode owns stable antibacterial activity and outstanding biocompatibility as well. We further elucidate the synergistic effect of the intracellular and extracellular ROS on the post‐charging antibacterial performance. In vivo study demonstrates that the MCO electrode could sufficiently kill bacterial and modulate local inflammatory status, consequently promoting the healing process of infected wound. The strategy provides insights into the development of efficient electrical antibacterial materials for wound infection therapy.

## Conflict of Interest

The authors declare no conflict of interest.

## Supporting information

Supporting Information

## Data Availability

The data that support the findings of this study are available in the supplementary material of this article.
