# Supplementary material for: Ultralow Charge Voltage Triggering Exceptional Post‐Charging Antibacterial Capability of Co3O4/MnOOH Nanoneedles for Skin Infection Treatment
Source: Adv Sci (Weinh). 2023 Jan 26;10(10):2207594. doi: 10.1002/advs.202207594 (PMC10074062; doi:10.1002/advs.202207594)
Supplement: Supplementary file 1 — Supporting Information [file ADVS-10-2207594-s001.pdf]

ADVANCED SCIENCE
Open Access

Supporting Information

for *Adv. Sci.*, DOI 10.1002/advs.202207594

Ultralow Charge Voltage Triggering Exceptional Post-Charging Antibacterial Capability of Co_3O_4/MnOOH Nanoneedles for Skin Infection Treatment

Xianshuo Cao, Zongshao Li, Fan Yang, Jinhao Xie, Xin Shi, Peiyan Yuan, Xin Ding and Xihong Lu**

Supporting Information

Ultralow Charge Voltage Triggering Exceptional Post-Charging Antibacterial Capability of Co_3O_4/MnOOH Nanoneedles for Skin Infection Treatment

Xianshuo Cao, Zongshao Li, Fan Yang, Jinhao Xie, Xin Shi, Peiyan Yuan, Xin Ding and Xihong Lu,**

Experimental Section

Materials: Carbon cloth was purchased from AvCarb (U.S.A.). $Co(NO_3)_2{\cdot}6H_2O$ was obtained from Aladdin (China). NH_4F was purchased from Macklin (China). $KMnO_4$, urea and ethanol were bought from Guangzhou Chemical Reagent Factory (China). Luria Bertani (LB) was obtained from BD (U.S.A.). *E. coli* (ATCC No.25922), *P. aeruginosa* (ATCC No. 27853), *K. pneumoniae* (ATCC No. 700603), *A. baumannii* (ATCC No. 1792) and human immortalized keratinocytes (HaCaT) cells were obtained from ATCC (U.S.A.). Glutaraldehyde, methylene blue (MB), iodonitrotetrazolium chloride (INT), 1,3-diphenylisobenzofuran (DPBF) and trichloroacetic acid were obtained from Macklin (China). Live/Dead BacLight bacterial viability staining kit was procured from Invitrogen (U.S.A.). DCFH-DA fluorescent probe and propidiumiodide (PI) were purchased from MCE (U.S.A.). Calcein AM (CAM) and DiSC3(5) were obtained from AAT Bioquest (U.S.A.). Polyethyleneimine (PEI) was obtained from Sigma-Aldrich (U.S.A.). NADH and CCK-8 assay kit were purchased from Beyotime (China). BALB/c mice were purchased from Sun Yat-Sen university laboratory animal center. All regents used in the experiments were analytical grade without further purification.

Preparation of electrodes: Firstly, Co(OH)F nanoneedles were prepared on carbon cloth by a facile hydrothermal method according to previous research. A piece of clean carbon cloth (2 cm x 2 cm) was immersed into ethanol and sonicated for 10 min. Then, 1.66 g $Co(NO_3)_2$, 0.42 g NH_4F and 1.71 g of urea were dissolved in 40 mL deionized (DI) water and stirred for 1 min. Subsequently, the mixed solution was transferred to a 25 mL Teflon-lined stainless steel autoclave and a piece of carbon cloth was then immersed into the solution. The autoclave was heated at 120 °C for 6 h. After naturally cooled down at room temperature, the sample was thoroughly washed with DI water and ethanol and further dried at 60 °C. The obtained Co(OH)F was heat treatment in air at 350 °C for 2 h to obtain Co_3O_4 nanoneedles (CO).

To obtain a MnOOH modified CO nanoneedles (MCO), the obtained CO was put into a Teflon-lined stainless steel autoclave containing a 0.03 M $KMnO_4$ solution, which was subsequently maintained at 160 °C for 1 h. Finally, the sample was repeatedly washed with DI water, and dried overnight at 60 °C to obtain MCO nanoneedles.

Materials characterization: The microstructures and compositions of samples were characterized by powder X-ray diffraction (XRD, D8 ADVANCE), field-emission scanning electron microscopy (FE-SEM, JSM-6330F), transmission electron microscopy (TEM, FEI Tecnai G^2 F30) equipped with an energy dispersive X-ray spectrometer (EDS), and X-ray photoelectron spectroscopy (XPS, Thermo VG).

Electrochemical measurements: The electrochemical performance of MCO were tested in a three-electrode system with the electrolyte of 0.1 M PBS aqueous. The synthesized materials were used directly as a working electrode, while a carbon rod and Hg/HgO electrode were utilized as counter electrode and reference electrode, respectively. The cyclic voltammetry (CV), and galvanostatic charge/discharge (GCD) electrochemical studies were recorded in an electrochemical workstation (CS150H). The electrochemical impedance spectroscopy (EIS), electrochemical surface areas (ECSA) and linear sweep voltammetry (LSV) curves were collected by the CHI760E electrochemistry workstation.

Calculation methods: DFT calculations were performed in the Vienna ab initio simulation package (VASP). The projector augmented wave (PAW) approach was used to describe the interaction between atomic cores and electrons. The exchange-correlation function was analysed by generalized gradient approximation (GGA) within Perdew-Burke-Ernzerhof (PBE). All electron plane-wave basis was set with an energy cutoff of 400 eV. Furthermore, the Brillouin-zone integrations used a (4×6×1) Monkhorst-Pack mesh. To eliminate the interaction among the slab, 10 Å vacuum layer was placed along the Z axis. The atomic relaxation was carried by using the conjugate gradient approach. The total energy convergence threshold was set at 1×10^{-5} eV, while the force convergence threshold was set at 0.01 eV/ Å.

Antibacterial activity of electrodes: Gram-negative bacteria including *E. coli*, *P. aeruginosa*, *K. pneumoniae*, *A. baumannii* were selected as the typical model bacteria for the evaluation of antibacterial activity of electrode. Briefly, bacteria were cultured in LB medium and left to grow at 37 °C for 18 h on a rotary shaker at 150 rpm. Afterwards, the bacterial suspension was further diluted with PBS to reach a concentration of 1×10^6 CFU mL^{-1}. The diluted bacterial solution was put into 6-well plate at 3 mL per cell. Subsequently, the electrodes after charging 30 min at 1.4 V were cleaned with a clean tissue paper to gently soak up the attached

solution, followed by immersing into above bacterial suspension. After incubation with electrodes for 5 min, the bacterial suspensions were diluted into 1×10^{4} CFU mL^{-1} and spread on agar culture plates to culture at 37 °C for another 15 h. Meanwhile, the group without electrical treatment was used as control. The antibacterial activity of electrodes against Gram-negative bacteria was determined by plate count methods.

SEM observation of bacterial morphology: The treated bacteria were centrifuged at 4000 rpm and immersed in 2.5% glutaraldehyde solution overnight at 4 °C. The fixed bacteria followed by washing with 0.1M PBS (pH 7.4) and dehydration with a series of graded ethanol solution (50%, 70%, 90%, 100%). Then, the bacteria were dried in 100% ethanol with liquid CO_2 at critical point. Finally, the treated bacteria were observed by SEM.

Live/dead bacterial assay: The Live/Dead BacLight bacterial viability staining kit was employed to evaluate the antibacterial properties of electrodes. Syto 9 with green fluorescence can stain all bacteria, regardless of live or dead, while PI only stains dead bacteria emitting red fluorescence. 3 μL of the equivalent mixture of Syto 9 and PI was added to 1 mL of the treated bacteria suspension and incubated for 15 min in the dark. Then, bacterial cultures were centrifuged and redispersed in 1 mL PBS solution. Finally, fluorescent images of bacterial samples were recorded by a confocal microscope (LSM 880 NLO with Airyscan, Zeiss).

Intracellular ROS determination: DCFH-DA fluorescent probe was applied to determine the bacterial intracellular ROS. Briefly, the treated bacterial suspension was incubated with DCFH-DA (final concentration of 10 μM) at 37 °C for 30 min. The fluorescence intensity of DCFH-DA was measured by a fluorescent spectroscopy (LS55, Perkin Elmer; E_x=488 nm, E_m=525 nm) to determine the amount of ROS generation. The DCFH-DA stained bacteria was also imaged using a confocal laser scanning microscope (LSM 880 NLO with Airyscan, Zeiss).

Extracellular ROS determination: The $^1O_2$ quantum yield was measured using DPBF as a $^1O_2$ trapping agent. The charged electrode was added into aqueous DPBF (final concentration of 100 μM), and incubated for 5 min. Then, the absorption intensity of DPBF was recorded by UV-vis spectrometer (Yoke Instrument T2602). MB was utilized to verify the generation of OH. Briefly, charged electrode was incubated with MB solution at a final concentration of 1 ug mL^{-1} for 5 min, followed by measuring the absorbance of MB by UV-vis spectrometer (Yoke Instrument T2602).

Bacterial cell membrane potential measurement: Bacterial suspension (1×10^{8} CFU mL^{-1}) was prepared and subjected to electrode treatment. After that, the treated bacteria were collected at time points of 5 min, and washed two times with PBS. Subsequently, DiSC3(5)

(working concentration of 5 μM) was added to the treated bacterial suspension. Upon thorough mixture and incubation at room temperature in the dark for 30 min. After rinsed by PBS, the fluorescence intensity was recorded on a fluorescence spectroscope (λ_{ex} = 660 nm). Note that PEI (500 μg mL^{-1}, Sigma) treatment was used as a positive control.

Assays of respiration in bacteria: The reduction of INT was utilized to explore the effect of electrodes on the bacterial respiration. Briefly, 10^6 CFU mL^{-1} of bacteria suspension was treated with electrode which had been charged at 1.4 V for 30 min. Then, 1 mM INT and 0.6 mM NADH were added as substrate and incubated for 1 h in the dark. INT reduction was stopped by addition of 5% trichloroacetic acid. Insoluble formazan was centrifuged at 13,000 g for 5 min. The absorbance of the supernatant at 485 nm was measured on a UV-vis spectrometer after adding 1 mL methanol (Yoke Instrument T2602).

Biocompatibility evaluation: *In vitro* biocompatibility of charged electrode was evaluated on human immortalized keratinocytes (HaCaT) cells. HaCaT cells were seeded in a 6-well plate with a density of 2 × 10^5 cells/well and incubated in a 37 °C humidified incubator (5% CO_2) for 24 h. Then, cell medium was removed, charged electrodes were immersed into fresh cell medium and incubated for 24 h. After that, the cells were transferred to fresh medium and the cell viability was determined by CCK-8 assay kit according product's instruction. The untreated HaCaT cells were used as control.

Live/dead cell staining assay: The live/dead cell staining assays were further studied to evaluate the cytotoxicity of electrodes. The cells were treated with the same protocols as above biocompatibility evaluation mentioned. Then, the cells were stained with CAM and PI according product's instruction. After being cultured for another 20 min, the cells were imaged by an inverted fluorescence microscope (Eclipse TS2-fl, Nikon).

***In vivo* antibacterial test:** All experiments were conducted in accordance with the rules of Animal Care and Use Committee of School of Pharmaceutical Sciences (Shenzhen), Sun Yat-Sen University (SYSU-YXYSZ-20210303). Six weeks old male Balb/c mice (20–25 g) were randomly divided into three groups (Control, CO, MCO). On the left rear back of each rat, a wound (≈1 cm^2) was created using a surgical scalpel. Subsequently, wounds were infected using 100 μL of *P. aeruginosa* bacterial suspension (10^9 CFU mL^{-1}). After 24 h infection, the mice were treated with the electrode for 5 min on days 1, 3, and 5, respectively. The digital images of wounds were taken on days0, 2, 4, 6and 10 to monitor the wound healing process. After sacrificing the animal on the 10th day, skin tissue samples were harvested on days 10 for hematoxylin and eosin (H&E), Masson's trichrome analyses. Besides, on day 2, 4, 6, the bacterial suspension obtained from the wounds was spread on agar plates to quantify the

viable bacterial cells.

Statistical analysis: Data were presented as mean or mean ± standard deviation. The statistical significance was determined using a t-test: ns: $p > 0.05$, *$P < 0.05$, *** $P < 0.01$.

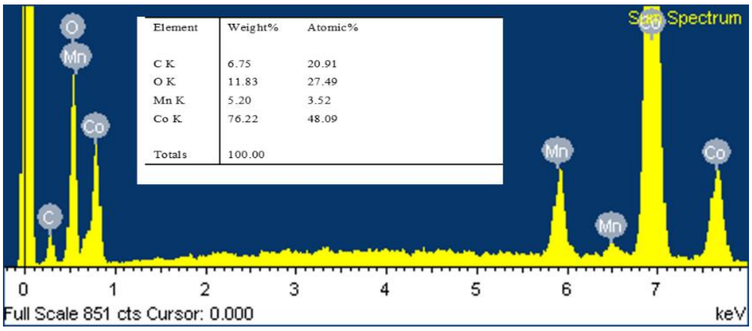

Figure S1. The EDS spectra of MCO.

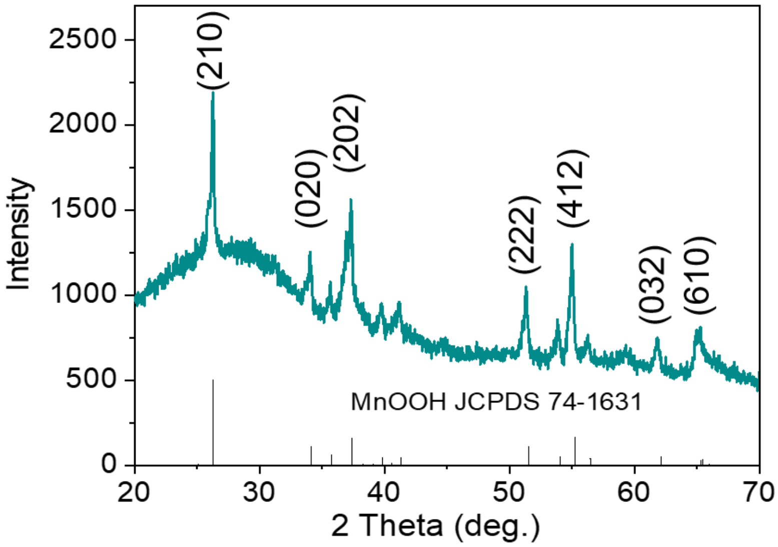

Figure S2. The XRD patterns of the precipitation generated during the MCO preparation.

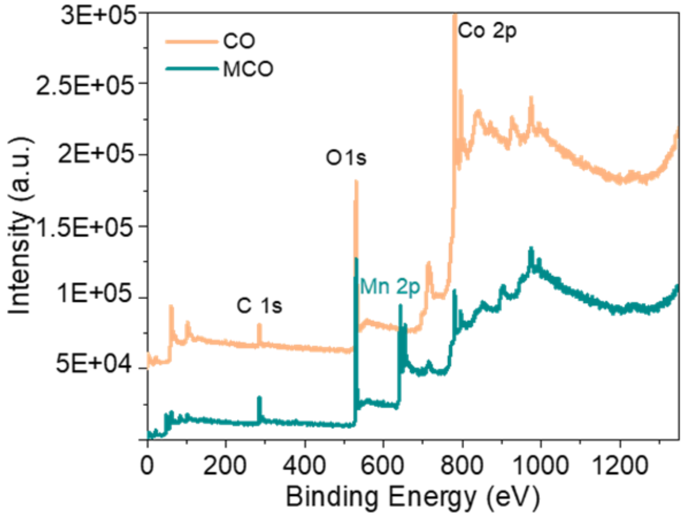

Figure S3. The broad XPS spectra for CO and MCO.

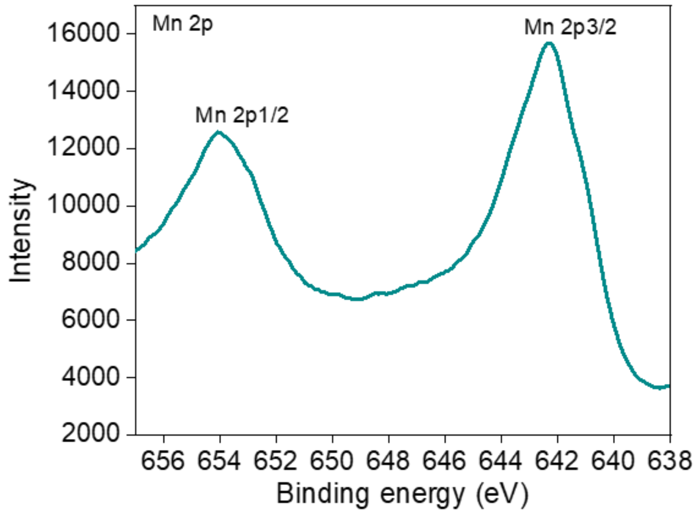

Figure S4. Mn 2p core-level XPS spectra for MCO.

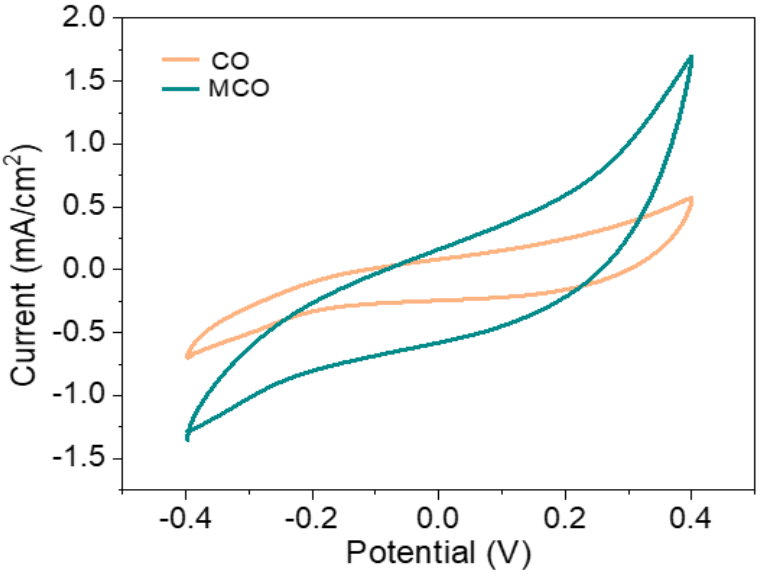

Figure S5. The CV curves of different electrodes.

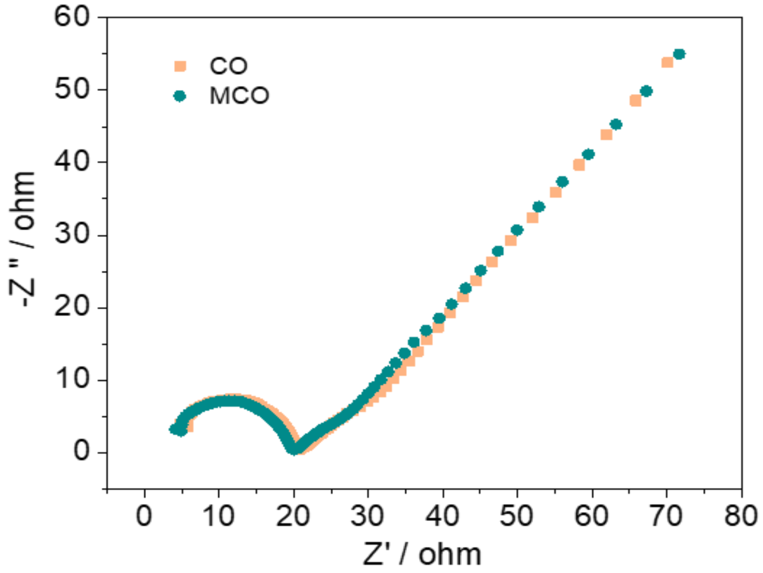

Figure S6. Nyquist plots of different electrodes.

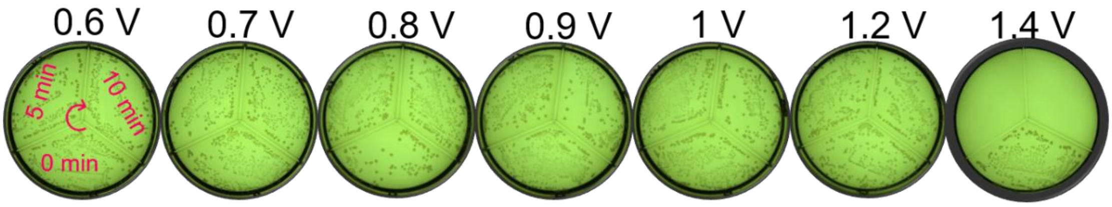

Figure S7. The post-charging antibacterial activity of MCO against *E. coli* prepared at different hydrothermal time.

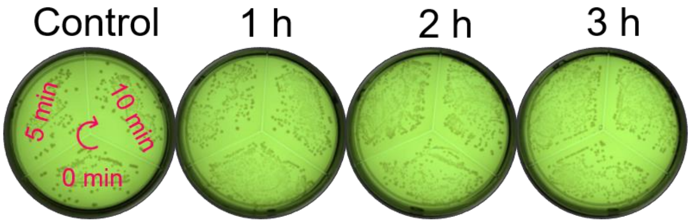

Figure S8. The post-charging antibacterial activity of MnOOH synthesized with different time against *E. coli* at 1.4 V for 30 min.

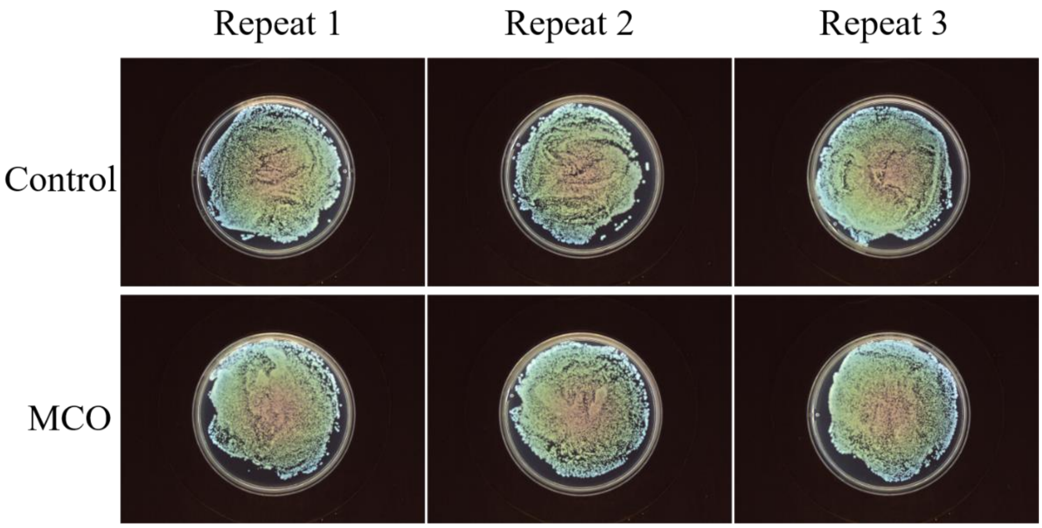

Figure S9. The antibacterial activity of uncharged MCO against *P. aeruginosa*.

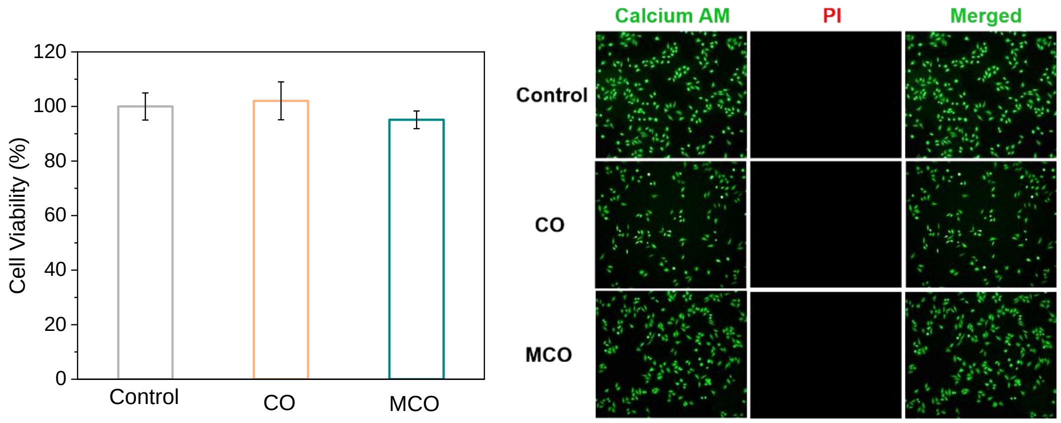

Figure S10. a) The relative viability of HaCaT cells after different treatments. b) HaCaT cells stained by calcein AM and PI after varied treatments.

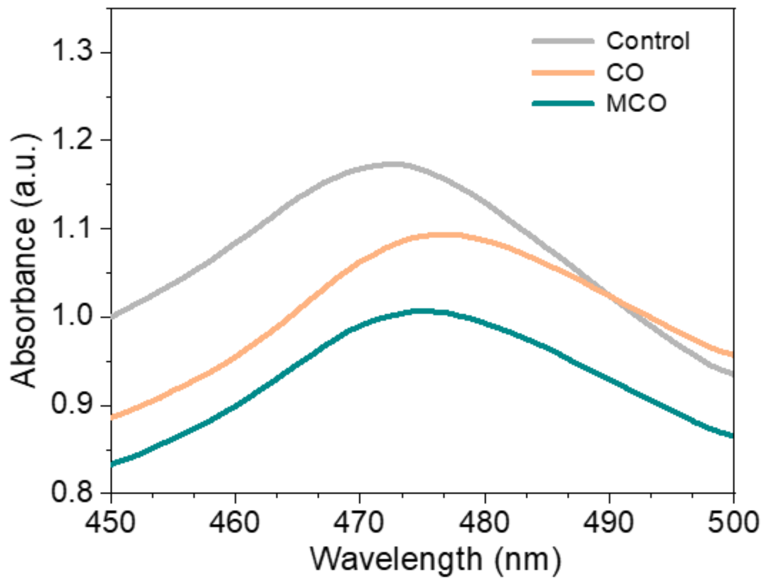

Figure S11. ROS ($^1O_2$) generation characterized by the UV absorption spectra of DPBF.

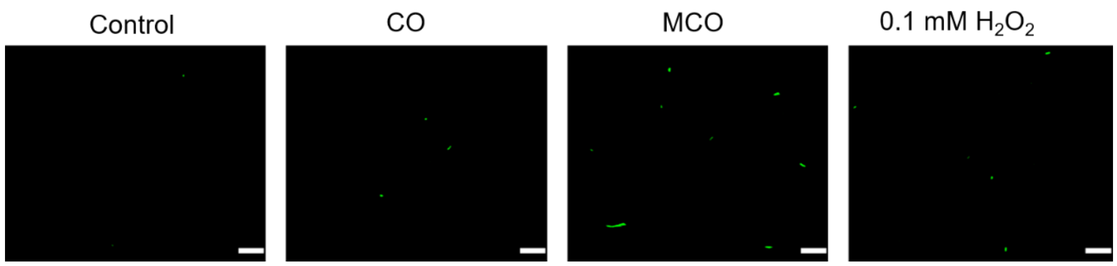

Figure S12. The confocal images of *E. coli* stained with intracellular fluorescent ROS probe DCFH-DA (scale bar: 10 μm).
